# Infiltration of immune cells into cancer cell/stroma cell 3D microtissues

**DOI:** 10.1186/2051-1426-3-S2-P75

**Published:** 2015-11-04

**Authors:** Stefan Koeck, Marit Zwierzina, Edith Lorenz, Gabriele Gamerith, Heinz Zwierzina, Arno Amann

**Affiliations:** 1 Department of Internal Medicine V, Medical University of Innsbruck, Innsbruck, Austria; 2 Department of Anatomy, Histology and Embryology, Medical University of Innsbruck, Innsbruck, Austria

## Background

The pharmacological manipulation of the interaction between the immune system, fibroblasts and cancer cells has been proven to be a key target for cancer therapy. Based on our previously developed co-culture system, this work aims to develop an innovative 3D cell culture system consisting of immune cells, fibroblasts and cancer cells and to investigate then influence of the tumor microenvironment on the infiltration of immune cells.

## Methods

3D tumor microtissues were cultivated using a “hanging drop” system. The human non-small cell lung cancer (NSCLC) cell lines A549 and Calu-6 with or without the human fibroblast cell line SV80 were incubated for 11 and 12 days to form microtissues. On day 10, peripheral blood mononuclear cells (PBMC) were added either with or without interleukin-2 (IL-2) for either 24 hours or 48 hours. The infiltration of PBMCs into the cancer cell and cancer cell/fibroblast microtissues was analyzed by immunohistochemistry (IHC). Quantification of the infiltrating subpopulations were performed with flow cytometry using surface staining on the cluster of differentiation (CD) 3, CD8, CD14, CD28, CD45, and CD45R0+.

## Results

Immunohistochemical staining revealed PBMC infiltration in cancer cell and cancer cell/fibroblast microtissues. In the A549/SV80 microtissues, PBMCs were located within the cancer cell compartment and hardly migrated into the fibroblast backbone of the microtissues. In the Calu-6/SV80 microtissues, the fibroblasts located at the margin of the microtissue seemed to prevent PBMCs from infiltrating into the cancer cell compartment. In both co-cultures and tri-cultures, infiltration of lymphocytes (CD14-) and monocytes (CD14+) was observed, with the proportion of lymphocytes prevailing the latter one. Under IL-2 stimulation, the percentage of CD14-lymphocytes and their subpopulations of CD3+CD8+ as well as CD45R0+ cells increased while the percentage of CD28+ cells decreased in both co- and tri-cultures.

## Conclusion

Our model shows that lymphocyte and activated effector T-cell infiltration into the cancer cell/fibroblast microtissues is stimulated by the addition of IL-2. We provide evidence that our 3D co-culture model is an effective tool to study interactions between cancer cells, fibroblasts and immune cells and to evaluate novel biomarkers for immunotherapy.

